# AN INTEGRATED ANALYSIS OF BETWEEN-COUNTRY DIFFERENCES IN DAILY COUPLING OF AGEIST ATTITUDES AND NEGATIVE AFFECT

**DOI:** 10.1093/geroni/igae098.1738

**Published:** 2024-12-31

**Authors:** Amit Shrira, Yuval Palgi, Reyyan Can, Anna Kornadt, Dwight Tse, Shevaun Neupert

**Affiliations:** Bar-Ilan University, Ramat Gan, HaMerkaz, Israel; Haifa University, Haifa, HaZafon, Israel; North Carolina State University, Raleigh, North Carolina, United States; University of Luxembourg, Esch-sur-Alzette, Luxembourg; University of Strathclyde, Glasgow, Scotland, United Kingdom; North Carolina State University, Raleigh, North Carolina, United States

## Abstract

Daily variations in subjective views of aging and their coupling with psychological concomitants are increasingly explored, yet potential cultural differences in these relationships have not been addressed. We, therefore, compared the daily coupling of ageist attitudes with negative affect (NA) across countries included in the Subjective AGES (Aging within Global Everyday Ecological Studies) project, encompassing 379 participants from the USA, UK, Germany, Israel, and Türkiye (ages ranged from 50 to 90; specific countries comprised 16% to 23% of the sample). Participants reported ageist attitudes and NA across 14 days. Within-person variability in ageist attitudes ranged from 15.5% (UK) to 53.7% (Türkiye) of the total variance. Overall, on days when participants had more ageist attitudes, they also reported higher NA. Cross-level interactions showed that the strength of ageist attitudes-NA coupling in the USA was moderate, while it was weaker in the UK and Germany and stronger in Israel and Türkiye. These findings suggest that the coupling of older adults’ daily ageist attitudes and NA varies across different countries. In European countries such as the UK and Germany, better access to healthcare services might mitigate the effects of ageist attitudes on NA. For Israel and Türkiye, the experience of a decline in social status due to a transition from a traditional and collectivist society to a modern and individualist one might increase the impact of views on aging. We will explore these explanations and future directions for the Subjective AGES project.

